# Identification of a cancer associated fibroblasts-related index to predict prognosis and immune landscape in ovarian cancer

**DOI:** 10.1038/s41598-023-48653-w

**Published:** 2023-12-07

**Authors:** Yingquan Ye, Shuangshuang Zhang, Yue Jiang, Yi Huang, Gaoxiang Wang, Mengmeng Zhang, Zhongxuan Gui, Yue Wu, Geng Bian, Ping Li, Mei Zhang

**Affiliations:** 1 Oncology Department of Integrated Traditional Chinese and Western Medicine, The First Affiliated Hospital of Anhui Medical University, Hefei, China; 2 Graduate School of Anhui University of Chinese Medicine, Hefei, China

**Keywords:** Cancer, Computational biology and bioinformatics, Biomarkers, Oncology

## Abstract

Cancer-associated fibroblasts (CAFs) play a role in ovarian cancer (OV) evolution, immunosuppression and promotion of drug resistance. Exploring the value of CAFs-related biomarker in OV is of great importance. In the present work, we developed a CAFs-related index (CAFRI) based on an integrated analysis of single-cell and bulk RNA-sequencing and highlighted the value of CAFRI in predicting clinical outcomes in individuals with OV, tumour immune microenvironment (TIME) and response to immune checkpoint inhibitors (ICIs). The GSE151214 cohort was used for cell subpopulation localization and analysis, the TCGA-OV patients as a training set. Moreover, the ICGC-OV, GSE26193, GSE26712 and GSE19829 cohorts were used for the validation of CAFRI. The TIMER 2.0, CIBERSORT and ssGSEA algorithms were used for analysis of TIME characteristics based on the CAFRI. The GSVA, GSEA, GO, KEGG and tumour mutation burden (TMB) analyses were used for mechanistic exploration. Additionally, the IMvigor210 cohort was conducted to validate the predictive value of CAFRI on the efficacy of ICIs. Finally, CAFRI-based antitumour drug sensitivity was analysed. The findings demonstrate that the CAFRI can served as an excellent predictor of prognosis for individuals with OV, as well as identifying patients with different TIME characteristics, differentiating between immune ‘hot’ and ‘cold’ tumour populations, and providing new insights into the selection of ICIs and personalised treatment regimens. CAFRI provides new perspectives for the development of novel prognostic and immunotherapy efficacy predictive biomarkers for OV.

## Introduction

According to the latest International Agency for Research on Cancer Global Cancer Burden Report, 313,959 new cases of ovarian cancer (OV) were reported worldwide in 2020, with 207,252 deaths due to OV^1^. OV is the third most common gynaecological tumour after cervix and corpus uteri cancer, while the mortality rate is the second highest^1^. Additionally, over 70% of ovarian cancers are not diagnosed until the disease has progressed to an advanced stage (stage III or IV) due to its insidious early symptoms^2–4^. Although recent improvements in diagnosis and treatment have reduced mortality in patients with OV^5^, the 5-year survival rate has not improved significantly and is only 46.2%^6^. Currently, widely accepted clinical predictors of OV prognosis include FIGO staging, histological type, tumour grade and size of residual tumour after surgery, among others^7,8^. Nevertheless, as OV is highly heterogeneous, exploring effective predictive biomarkers at the molecular level is critical for prognostic and personalised therapeutic decision-making in OV.

The ‘seed and soil’ theory has led to the recognition that the tumour microenvironment (TME) plays an important role in tumour development^9^. Additionally, a growing number of studies have confirmed interactions between different cellular components of the TME are critical for tumourigenesis, evolution, metastasis and therapeutic efficacy^10–12^. Cancer-associated fibroblasts (CAFs) are an essential ingredient of TME^13,14^, which crosstalk extensively with cancer cells to regulate cancer progression and therapeutic response^15^ and are involved in negative immune regulation of tumours and drug resistance^16,17^. Abundant stromal interstitium is an important feature of OV^18^. Accumulating evidence suggests that CAFs are one of the most crucial members of the fibroproliferative mesenchyme in OV, regulating the evolution and therapeutic response of OV^19,20^. Furthermore, CAFs can promote immunosuppression and macrophage polarization in OV through released prostaglandins^21^ and have been suggested as potential targets for OV therapy^22^. Therefore, the potential value of CAFs-related biomarkers in OV deserves further exploration.

Technologies such as high-throughput sequencing, particularly single-cell RNA sequencing (scRNA-seq), can be used to analyse the status of TME at the single-cell level and to assess the differences between different cell subpopulations in TME^23^. In this study, we performed an integrated analysis of single-cell and bulk RNA-sequencing based on OV samples to develop a CAFs-related index (CAFRI) and highlight its advantages in predicting patient clinical outcomes, tumor immune microenvironment (TIME) and efficacy of immune checkpoint inhibitors (ICIs). These results provide insights into the exploring of OV biomarkers and the selection of individualised treatment regimens.

## Methods

### Downloading and analyzing data

**Figure 1:**
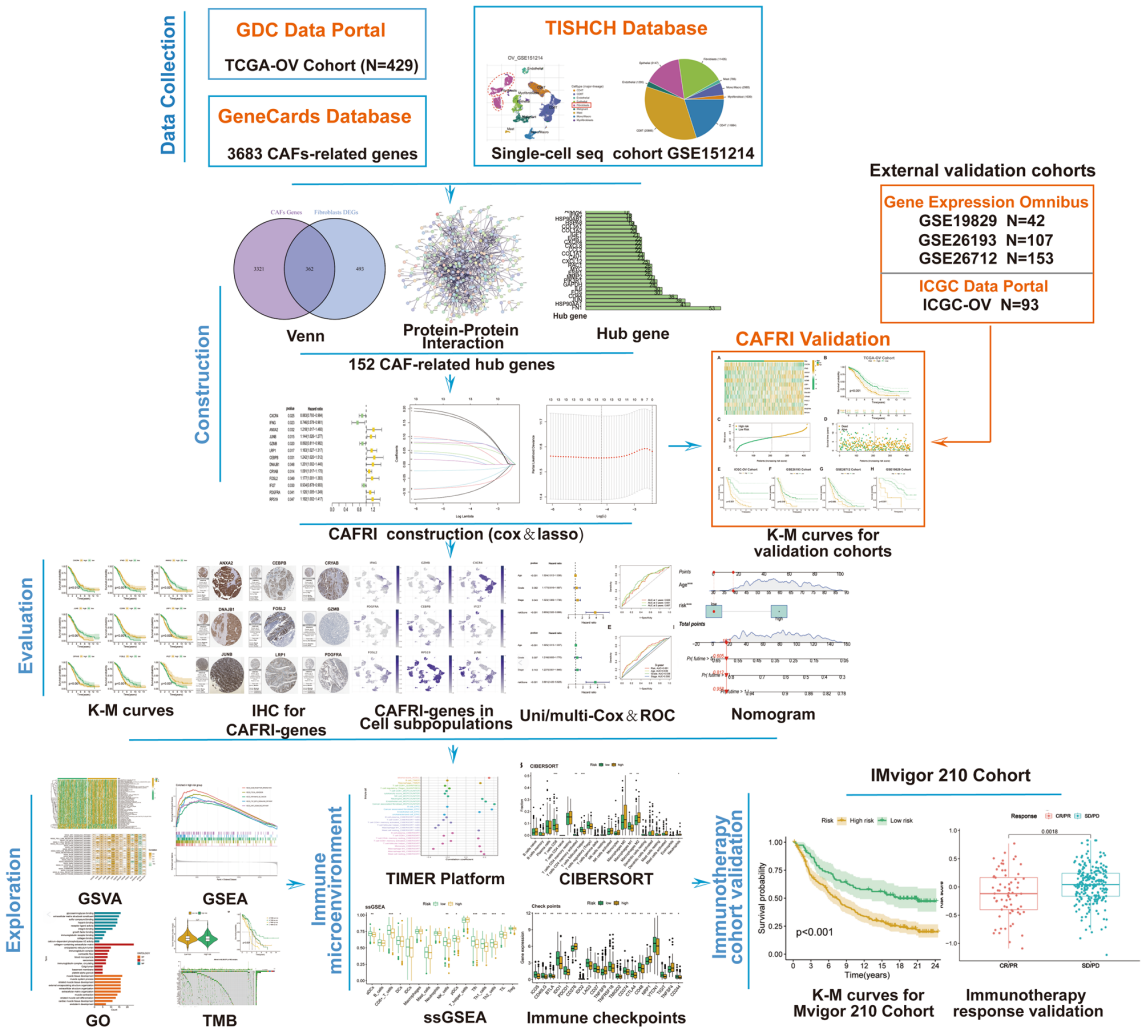
Workflow of the present research. A total of 3683 CAFs-related genes were included in the study. Of these, 152 CAF-related genes were identified as hub genes associated with CAF in the OV. Cox and LASSO regression ultimately identified 13 genes involved in the construction of the CAFs-related index (CAFRI). The ICGC-OV, GSE26193, GSE26712 and GSE19829 cohorts were used for the validation of CAFRI. The K-M, ROC curves and Cox regression were employed for the evaluation of the Index. The GSVA, GSEA, GO, KEGG and tumour mutation burden (TMB) analyses were used for mechanistic exploration. Furthermore, the TIMER, CIBERSORT and ssGSEA algorithms were used for analysis of immune landscape based on the CAFRI. Finally, the IMvigor210 cohort was conducted to validate the predictive value of CAFRI on the efficacy of immunotherapy.

The workflow of this study is shown in Fig. 1. Inclusion criteria for the training and validation cohorts of this study were cases that contained both transcriptomic data and survival information. The RNA-seq data, simple nucleotide variant (SNV) data and clinical parameters for OV in the TCGA-OV training cohort (n = 429) were downloaded from TCGA (https://portal.gdc.cancer.gov/repository). Perl version 5.32.1.1 was employed to obtain the mutation data for each sample based on SNV data. Transcriptome and clinical data for the GSE26193 (n = 107), GSE26712 (n = 153) and GSE19829 (n = 42) validation cohorts were downloaded from the GEO database (https://www.ncbi.nlm.nih.gov/). Another independent validation cohort (ICGC-OV) (n = 93) were obtained from the ICGC data portal (https://dcc.icgc.org/releases/current/Projects/OV-AU). The scRNA-seq dataset for the GSE151214 cohort (n = 8) were downloaded from the Tumor Immune Single-cell Hub (TISCH) platform (http://tisch.comp-genomics.org/), and the cell annotations were based on the major-lineage entry and the existing classical cell marker annotations in the TISCH^24^. The immune checkpoint inhibitor treatment cohort IMvigor210 (n = 298) were obtained from a previous study^25^. Immunohistochemical images of CAFRI were obtained from the Human Protein Atlas (https://www.proteinatlas.org). The Human Gene Database (https://www.genecards.org/) was used to retrieve the genes associated with CAFs^26^.

### Identification of CAFs-related hub genes

Differentially expressed genes (DEGs) (|fold change (FC)|> 1.5, false discovery rate (FDR) < 0.05) were identified between CAFs and other cell subtypes in the GSE151214 set, which contains DEGs that are up-regulated and down-regulated expression in CAFs. Through Venn diagrams we obtained overlapping genes for CAFs-related genes and DEGs. The STRING platform (Version11.5, https://cn.string-db.org/) was further utilized to determine the interaction relationships between the proteins encoded by the overlapping genes and to map the network relationships and explore the core genes. Genes with greater than 5 adjacent nodes in interaction network were included in subsequent analysis.

### Construction of a CAFRI in OV

We first performed univariate Cox regression analysis of hub genes in the TCGA-OV cohort to identify genes associated with risk. To prevent overfitting, a least absolute shrinkage and selection operator (LASSO) analysis was performed on risk-associated genes to identify the optimal prognostic genes involved in the construction of the CAFI^27^. The formula for the CAFRI score is as follows: CAFRI score = Expression (CAFRI GENE1) × Coefficient (CAFRI GENE1) + Expression (CAFRI GENE2) × Coefficient (CAFRI GENE2) + **⋯** + Expression (CAFRI GENEn) × Coefficient (CAFRI GENEn). The index score was calculated based on the expression of genes in CAFI and LASSO regression coefficients; higher regression coefficients of the genes in CAFRI suggest a higher risk for the genes, and therefore higher CAFRI scores represent a poorer prognosis for the patients. Individuals with OV in the training set were then categorised into high- and low-risk subgroups by risk stratification based on the median index score.

### Validation of the CAFRI

The ‘survivor’ and ‘survminer’ packages were applied to the Kaplan–Meier (K–M) analysis of the TCGA-OV cohort and K-M curves were plotted. The R package ‘pheatmap’ was used for gene expression heat mapping in the CAFRI. Furthermore, the CAFRI were also applied to the ICGC-OV, GSE26193, GSE26712 and GSE19829 cohorts for K-M survival validation. Cox analysis was utilised to determine the prognostic predictive specificity of the CAFRI. To further assess the prognostic predictive efficacy of the CAFRI, the R packages ‘survival’, ‘survminer’ and ‘timeROC’ were used for receiver operating characteristic (ROC) analysis and compared with AUC values for age, sex and stage.

### Nomogram construction

Based on Cox analysis, a nomogram for 1-, 3-, and 5-year overall survival (OS) were developed using the R packages ‘rms’, ‘survival’ and ‘regplot’. Hosmer–Lemeshow calibration curves were plotted to confirm the correlation between the actual results and the predicted values.

### Enrichment pathway analysis

To further analyse the biological functions among different risk groups identified based on CAFRI, we further performed functional enrichment analyses. GSVA is a special type of gene set enrichment method^28^. We explored the enrichment of KEGG pathways in different risk groups by GSVA and mapped the enrichment heatmap. This process was implemented with the R packages ‘limma’^29^, ‘GSVA’, ‘GSEABase’ and ‘pheatmap’. In additiona, we investigated the association between CAFRI gene expression and signaling pathways by R packages ‘limma’, ‘GSEABase’, ‘GSVA’, ‘reshape2’ and ‘ggplot2’.

GSEA an algorithm that calculates whether a predefined set of gene sets has a statistically significant difference between two biological traits or states^30^. GSEA was used to obtain the functional pathways that were enriched in the different subgroups and to visualise the five most enriched functions. The process was implemented through ‘limma’, ‘org.Hs.eg.db’, ‘DOSE’, ‘clusterProfiler’^31^ and ‘enrichplot’. Finally, the ‘limma’ was used to identify DEGs between the two risk subgroups (|FC|> 2, FDR < 0.05) and further gene ontology (GO) analysis was conducted to investigate the enrichment of DEGs in cellular component, molecular function and biological process.

### Tumour mutation burden (TMB) analysis

The SNV data downloaded was processed utilizing strawberry-perl to obtain a matrix of TMB data. The ‘limma’ and ‘ggpubr’ were performed to compare TMB levels between different risk subgroups and the results were visualised. K-M method was performed to investigate the prognostic differences between the various TMB /risk subgroups. Furthermore, the 30 genes with the highest mutation frequencies were extracted and their mutation waterfalls were plotted using the visualisation tool ‘maftools’^32^.

### CAFRI-based landscape analysis of TIME

TIMER is a comprehensive resource that contains six distinct algorithms for inferring the extent of immune cell infiltration across diverse cancer types^33,34^. Correlations between immune cell levels and CAFRI scores were determined and visualised by analysing tumour infiltrating immune cell dataset from TIMER 2.0 (http://timer.comp-genomics.org/). The above process was performed by ‘ggplot2’, ‘tidyverse’, ‘ggtext’, ‘scales’ and ‘ggpubr’ packages. GSEA has been shown to classify genomes that share common biological features^30^. Here, the single-sample GSEA (ssGSEA) algorithm^35^ was conducted utilizing the ‘GSEABase’ and ‘GSVA’ packages to estimate the levels of immune cell infiltration in each tumour sample in TCGA-OV to obtain the immune cell and immune-related function scores. The ‘ggpubr’ and ‘reshape2’ were were applied to realise the visualisations. Hyperactivation of immune checkpoints is important for tumour immune escape^36^. We also investigated the expression levels of different immune checkpoints between the different subgroups. Furthermore, the ICIs treatment cohort IMvigor210 was utilised to speculate on immunotherapy response in CAFRI.

### Drug response prediction

The R package ‘pRRophetic’ has been used to impute the sensitivity of antitumour drugs from transcriptome expression levels^37^. This study explores the half-maximal inhibitory concentrations (IC50) of different antitumour agents in the two risk groups by ‘pRRophetic’ to investigate the role of CAFRI in guiding the individualised treatment of individuals with OV.

## Results

### CAFs in OV

**Figure 2:**
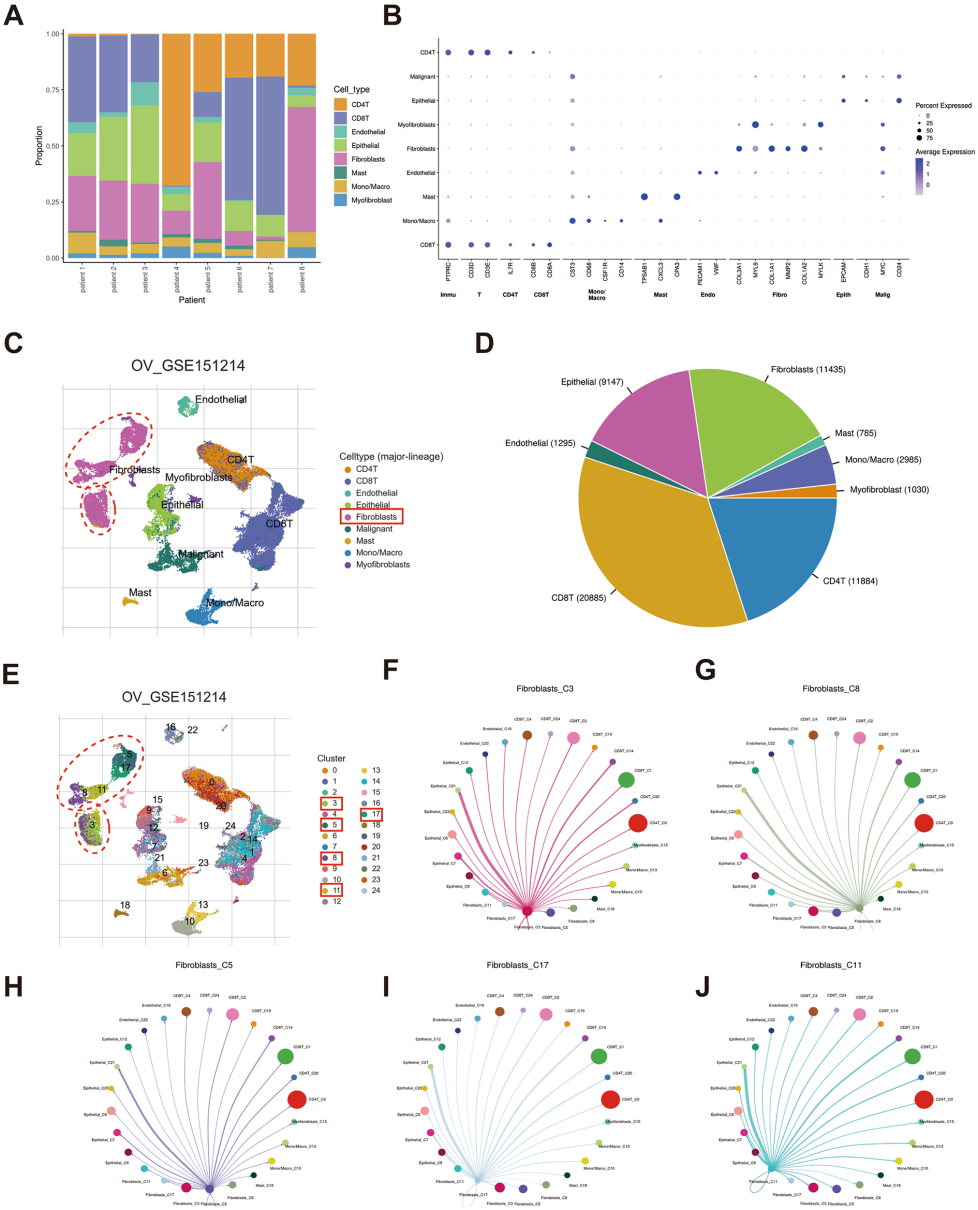
Identification of CAFs in OV. (**A**) Proportion of distinct cells in eight OV samples from the GSE151214. (**B**, **C**) All cells were categorised into nine types (CD4+ T cells, CD8+ T cells, epithelial cells, endothelial cells, fibroblasts, malignant cells, mast cells, monocytes and fibroblasts). (**D**) Proportion of distinct cell types in the total sample. (**E**) The identified cell clusters (n = 24) in OV tissues in the GSE151214 cohort, where clusters 3, 5, 8, 11 and 17 were fibroblasts. (**F**–**J**) The interrelationships between the different clusters of fibroblasts and other cells.

Cells in the GSE151214 cohort were classified into nine types according to the TISCH annotations, including CD4 + T cells, CD8 + T cells, epithelial cells, endothelial cells, fibroblasts, malignant cells, mast cells, monocytes and fibroblasts (Fig. 2B, C). The strip graph reveals the proportion of different cells in GSE151214 cohort (Fig. 2A). And the pie chart shows the ratio of distinct cell types in the total sample, with CD8+ T cells being the highest and fibroblasts in third place (Fig. 2D). Additionally, analysis of scRNA-seq data from the GSE151214 cohort identified 24 cell clusters, of which clusters 3, 5, 8, 11 and 17 were fibroblasts (Fig. 2E). Furthermore, network diagrams showed the cellular communication between the different clusters of fibroblasts and other cells (Fig. 2F–J), suggesting extensive interaction among CAFs and different cells in the TME.

### Determination of hub CAFs-associated genes in OV

**Figure 3:**
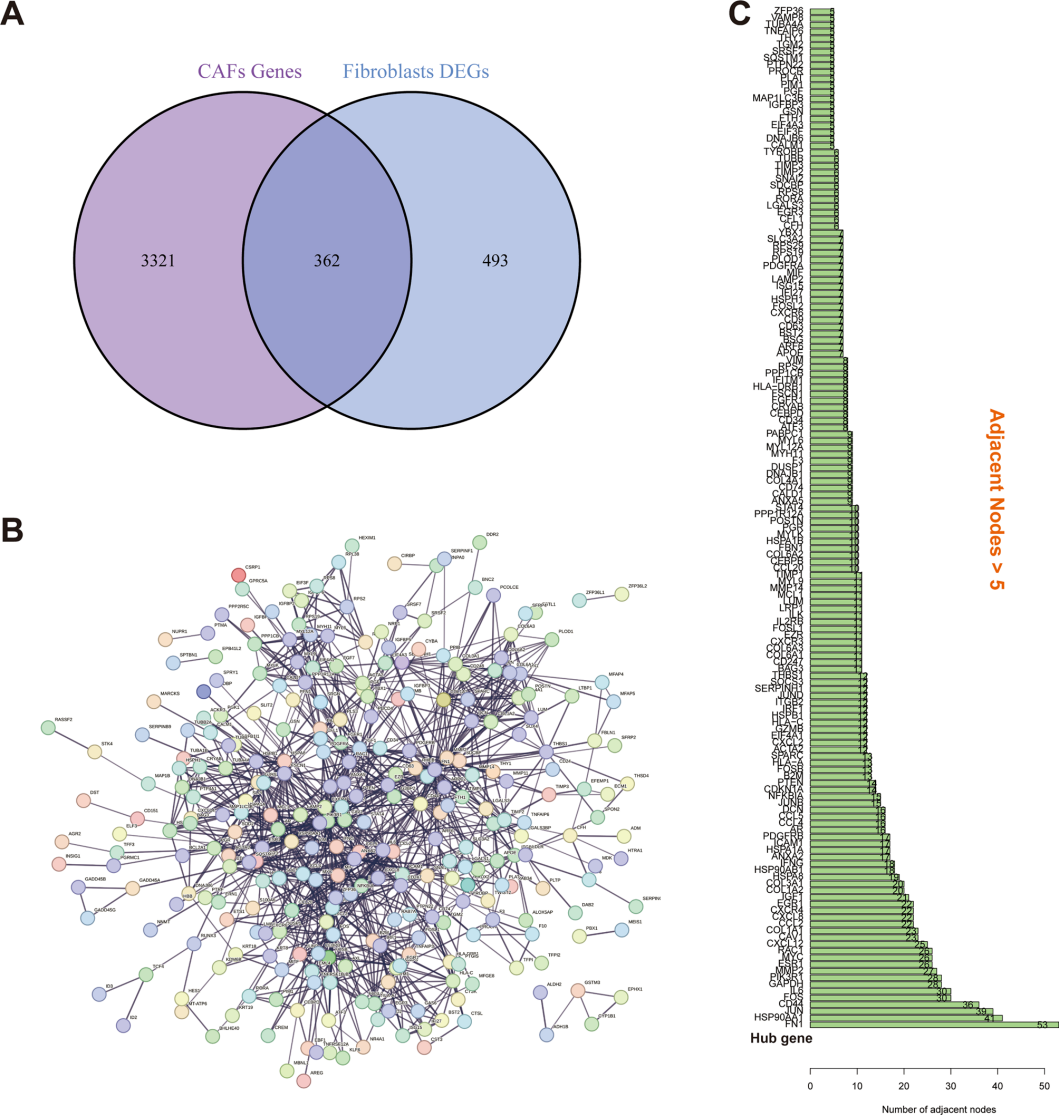
Identification of hub CAFs-related genes. (**A**) Venn diagram identified 362 CAFs-related genes in OV. (**B**) Protein–protein interaction (PPI) network plot for the 362 CAFs-related genes. (**C**) The 152 genes having greater than 5 adjacent nodes in their neighbourhood.

The Venn diagram showed 855 genes differentially expressed across fibroblasts and other cells, which were further crossed over with the CAFs associated genes to yield 362 overlapping genes (Fig. 3A). The interactive network plots showed the interactions between the proteins encoded by the 362 overlapping genes (Fig. 3B), with 152 genes having greater than 5 adjacent nodes in their neighbourhood. These genes were identified as CAF-related hub genes in OV (Fig. 3C).

### Establishment of a CAFRI based on Cox and LASSO

**Figure 4:**
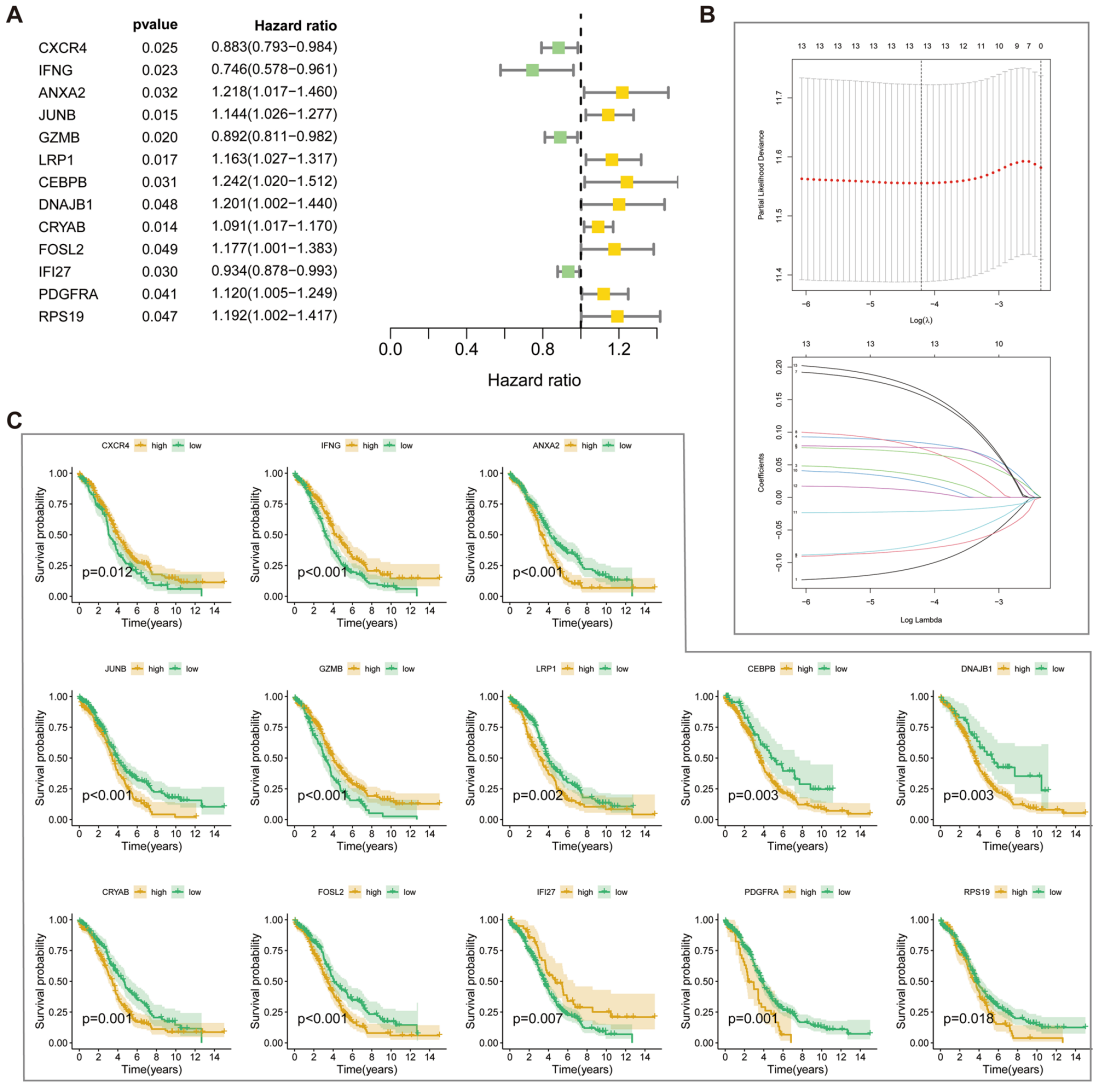
Establishment of a CAFRI in OV. (**A**) Univariate Cox analysis identifies 13 CAFs genes associated with prognosis of OV. (**B**) LASSO regression analysis identifies 13 CAF-related genes involved in CAFRI construction. (**C**) Kaplan–Meier curves for the 13 CAFRI genes in the training set.

**Figure 5:**
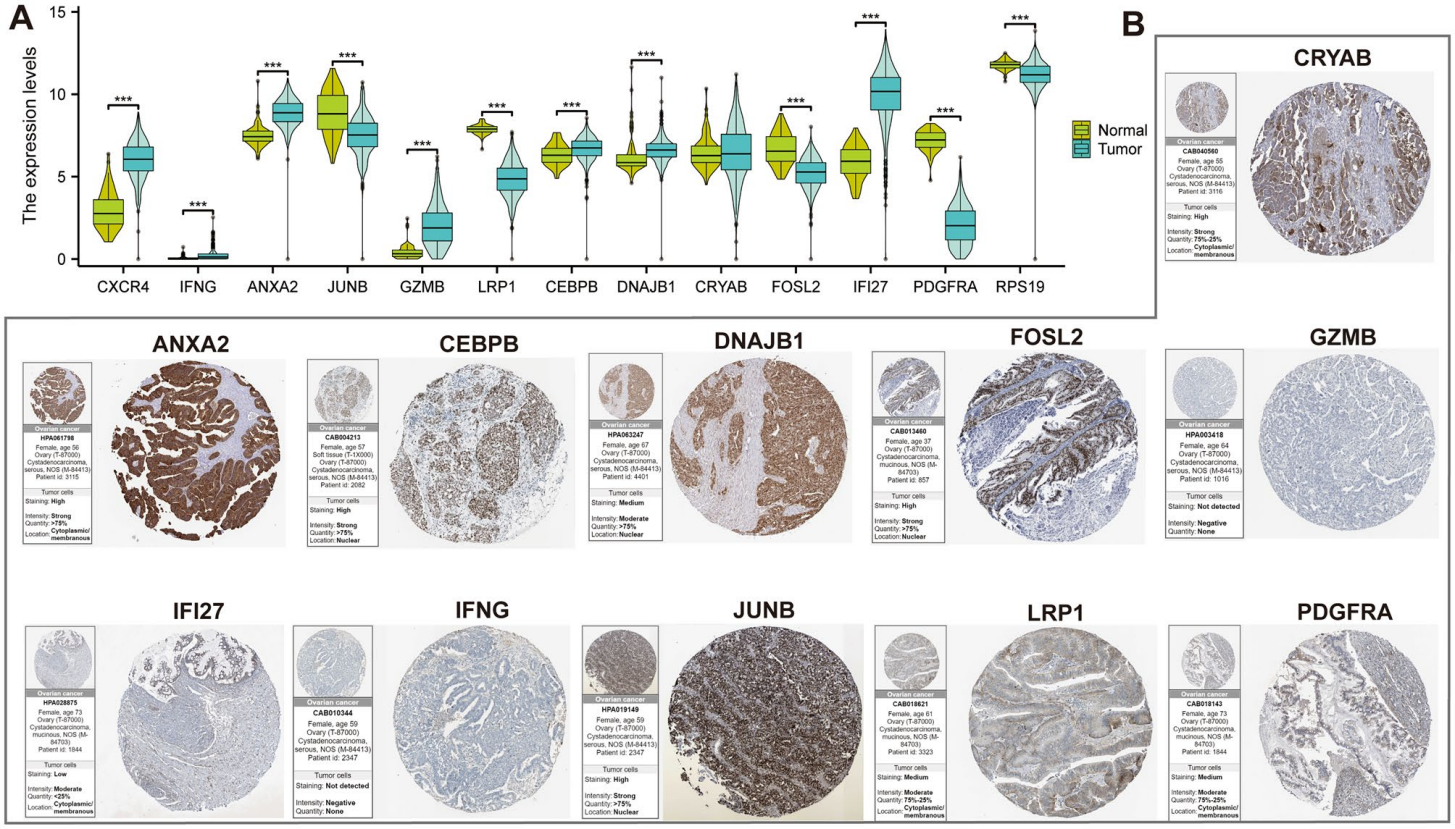
CAFRI-related genes in the OV. (**A**) Differential expression of the 13 CAFRI-related genes in tumor and normal tissues of the TCGA-OV cohort. (**B**) Immunohistochemical images of CAFRI-related proteins in OV from the HPA portal.

We performed univariate Cox analysis on 152 hub genes and obtained 13 genes associated with disease risk, of which nine were risk factors and and four were protective factors (Fig. 4A). The machine learning LASSO algorithm further identified the optimal genes involved in CAFRI construction (Fig. 4B) (Table 1). Additionally, K-M curves for CAFRI genes in the training set showed *CXCR4*, *IFNG*, *GZMB* and *IFI27* as good prognosis genes, while the rest were poor prognosis genes, a result that was validated against the Cox regression results described above (Fig. 4C). Further analysis showed that *ANXA2*, *DNAJB1*, *CEBPB*, *RPS19* and *JUNB* were expressed at high levels in fibroblasts (Fig. S1). Furthermore, CAFRI-related genes were differentially expressed in OV tumour tissues, with the exception of *CRYAB* (Fig. 5A). Immunohistochemical images show the expression of CAFRI-related proteins in the HPA database (Fig. 5B). Based on the regression coefficients and expression of the CAFRI-related genes, we obtained a CAFRI score for each patient. CAFRI score = Expression (*RPS19*) * (0.164449) + Expression (*CEBPB*) * (0.156762) + Expression (*JUNB*) * (0.083987) + Expression (*LRP1*) * 0.076594) + Expression (*DNAJB1*) * (0.075415) + Expression (*CRYAB*) * (0.067759) + Expression (*ANXA2*) * (0.033018) + Expression (*FOSL2*) * (0.023497) + Expression (*PDGFRA*) * (0.009336) − Expression (*IFI27*) * (0.021267) − Expression (*GZMB*) * (0.072410) − Expression (*IFNG*) * (0.079514) − Expression (*CXCR4*) * (0.105466). Finally, all patients in the training and validation sets were risk stratified based on the median CAFRI score of patients in the training set.

**Table 1:** CAFs-related index in OV.

CAFs gene	Coefficient	HR	HR (95%CI)	*p*-value
ANXA2	0.033018	1.218	1.017–1.460	0.032
CEBPB	0.156762	1.242	1.020–1.512	0.031
CRYAB	0.067759	1.091	1.017–1.170	0.014
CXCR4	− 0.105466	0.883	0.793–0.984	0.025
DNAJB1	0.075415	1.201	1.002–1.440	0.048
FOSL2	0.023497	1.177	1.001–1.383	0.049
GZMB	− 0.072410	0.892	0.811–0.982	0.020
IFI27	− 0.021267	0.934	0.878–0.993	0.030
IFNG	− 0.079514	0.746	0.578–0.961	0.023
JUNB	0.083987	1.144	1.026–1.277	0.015
LRP1	0.076594	1.163	1.027–1.317	0.017
PDGFRA	0.009336	1.12	1.005–1.249	0.041
RPS19	0.164449	1.192	1.002–1.417	0.047

CAFs, cancer-associated fibroblasts; HR, hazard ratio.

### Validation of the CAFRI in OV

**Figure 6:**
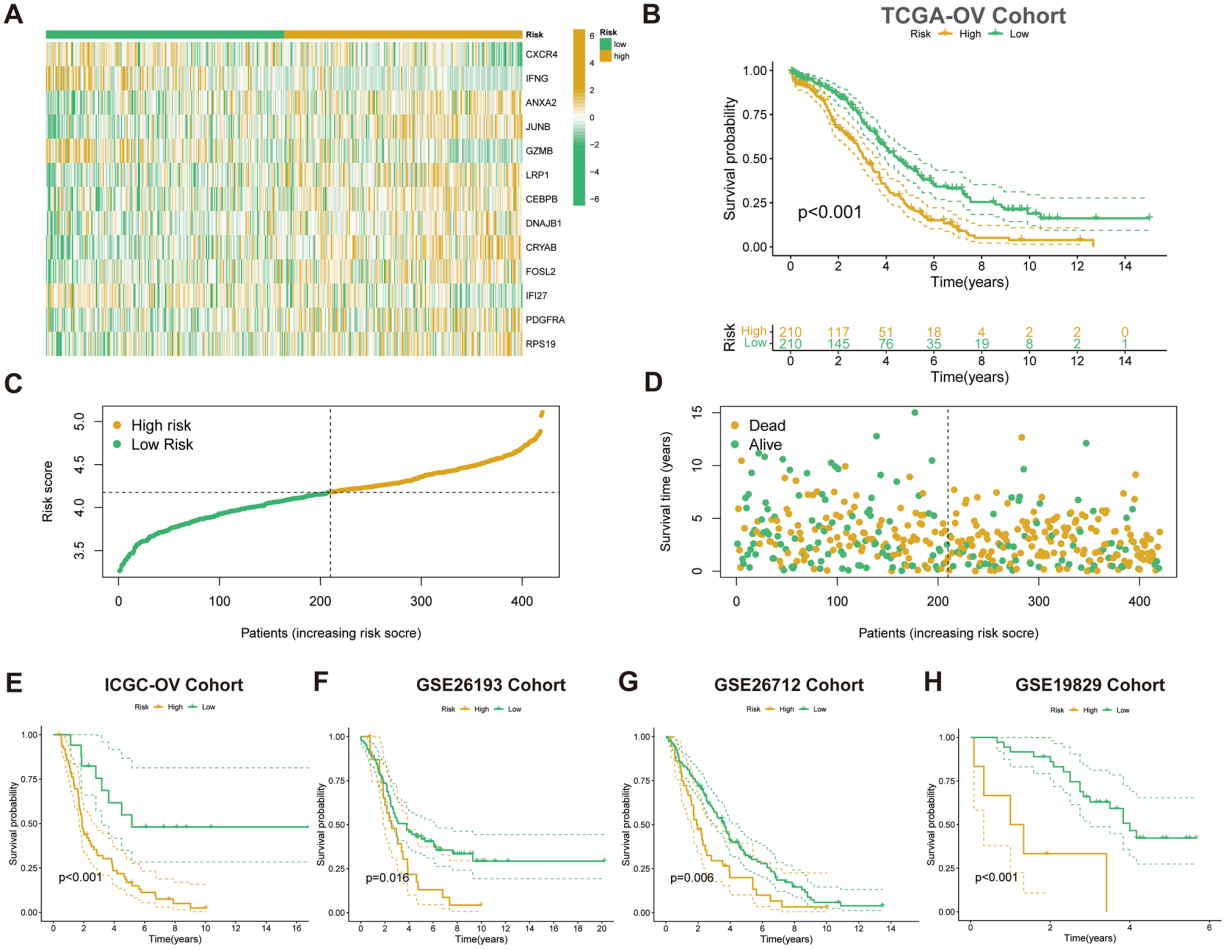
Validation of the CAFRI in OV. (**A**) Expression heat map of CAFRI-related genes in the high and low risk subgroups of the TCGA-OV set. (**B**) K–M curve for OS in the TCGA-OV set. (**C**, **D**) Risk score and survival state in the TCGA-OV set. (**E–H**) Kaplan–Meier curves for CAFRI in the ICGC-OV, GSE26193, GSE26712 and GSE19829 validation cohorts.

The expression heat map of CAFRI-related genes in the TCGA-OV cohort revealed the expression levels of four genes with a favourable prognosis were lower in the high-risk subgroup, whereas nine risk factors were highly expressed in the high-risk subgroup (Fig. 6A). K–M survival curves revealed that the prognosis of the high-risk OV population, as determined by CAFRI, was significantly worse than that of the low-risk subgroup (*p* < 0.001) (Fig. 6B). Meanwhile, individuals from the low-risk subgroup already exhibit a better prognosis with statistically lower risk and fewer deaths (Fig. 6C, D). Subsequently, CAFRI scores were obtained for the four independent validation cohorts (ICGC-OV, GSE26193, GSE26712, and GSE19829) according to the CAFRI formula, and all patients were risk-stratified according to the optimal cutoff value of the K–M method for each cohort. K–M curves showed that the survival of the low-risk group was significantly better than that of the high-risk group in all validation cohorts (Fig. 6E–H).

### Assessment of the CAFRI and nomogram

**Figure 7:**
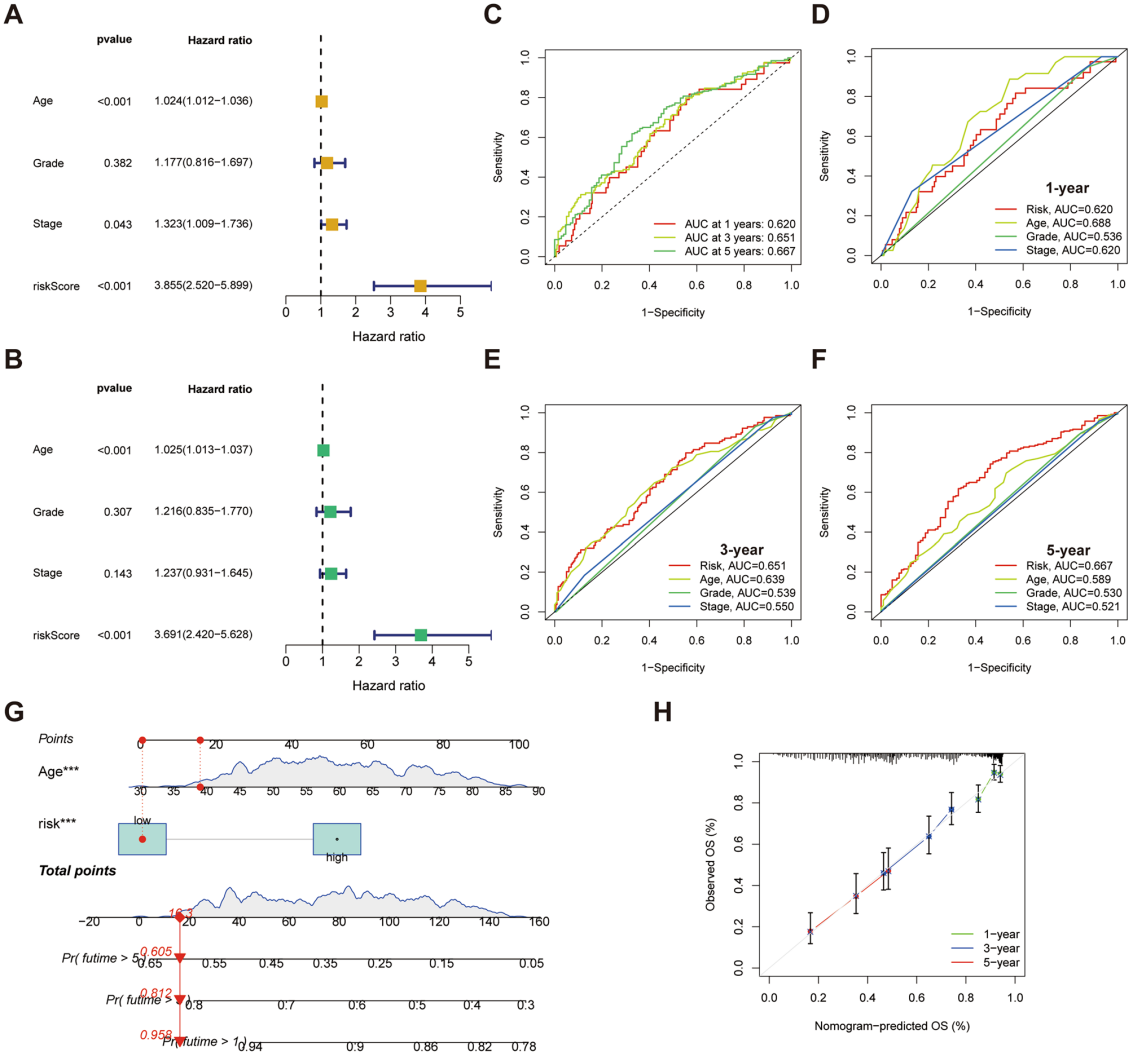
Assessment of the CAFRI and nomogram in OV. (**A**, **B**) Univariate and multivariate Cox regression suggests CAFRI and age are independent prognostic variables for OV. (**C**) ROC curves for the CAFRI in the training set at 1, 3, and 5 years. (**D**–**F**) ROC curves for age, grade and stage at 1, 3, and 5 years. (**G**) Nomogram including age and CAFRI risk status for predicting OS of OV. (**H**) The calibration curves of the nomogram at 1, 3, and 5 years. *P < 0.05, **P < 0.01, and ***P < 0.001.

The results of the univariate and multivariate Cox regression showed that the CAFRI-based risk score and age were independent prognostic predictors of OV (*P* < 0.001) (Fig. 7A, B). Additionally, ROC curves showed that CAFRI had AUC values of 0.620, 0.651 and 0.667 at 1-, 3- and 5- years, while age had AUC values of 0.688, 0.639 and 0.589, respectively (Fig. 7C–F). Based on the multivariate Cox regression, the age and CAFRI-based risk status were included in the nomogram construction. We estimated the OS rates for a patient with low-risk and 39-year-old at 1-, 3- and 5-year to be 0.958, 0.812, and 0.605 (Fig. 7G). The calibration curves showed a high agreement between the predicted outcomes based on the nomogram and the actual survival rates of OV patients (Fig. 7H).

### CAFRI-based enrichment analysis

**Figure 8:**
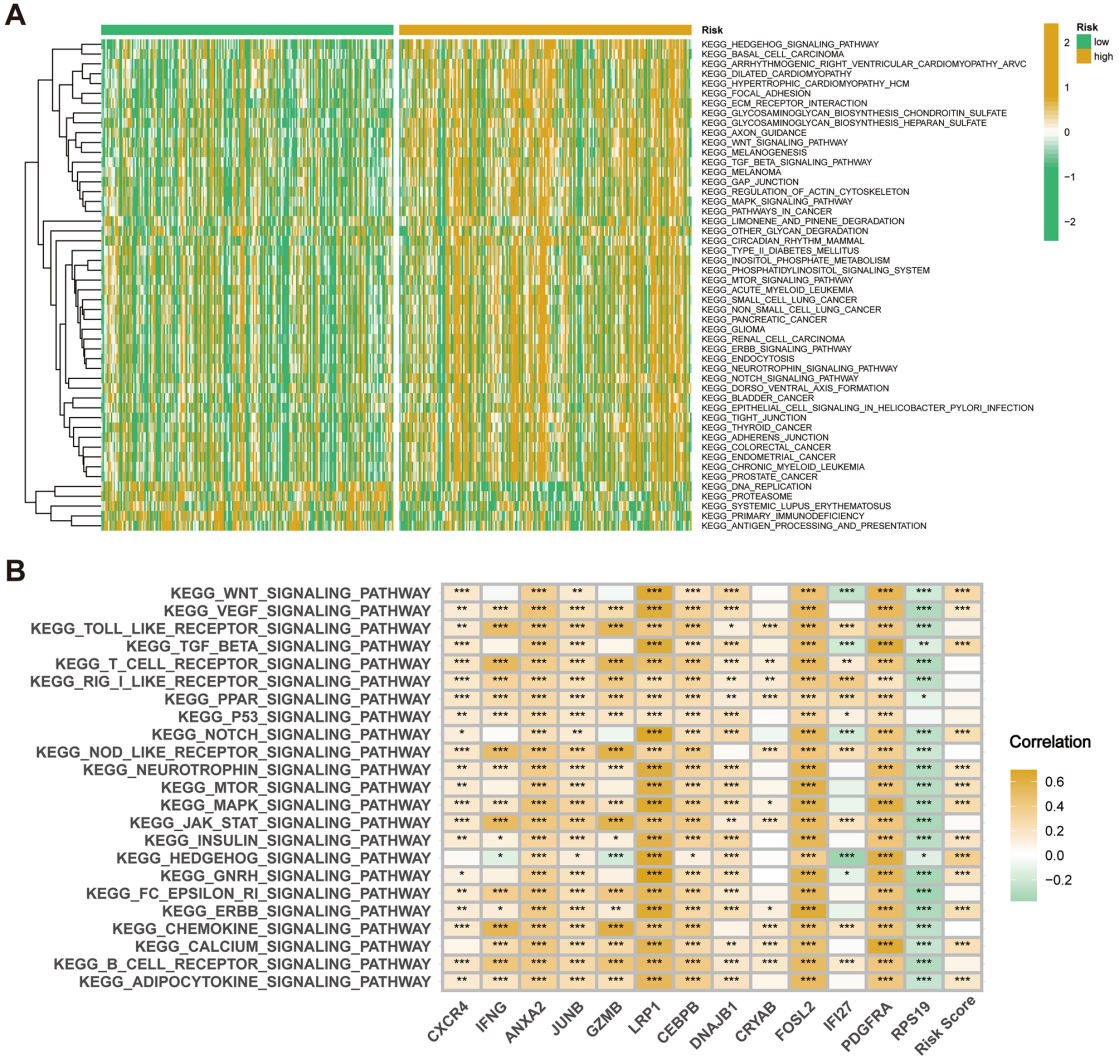
CAFRI-based GSVA in the TCGA-OV cohort. (**A**) GSVA identifies pathways enriched in high and low risk subgroups. (**B**) Correlation between the CAFRI-associated genes and signalling pathways.

**Figure 9:**
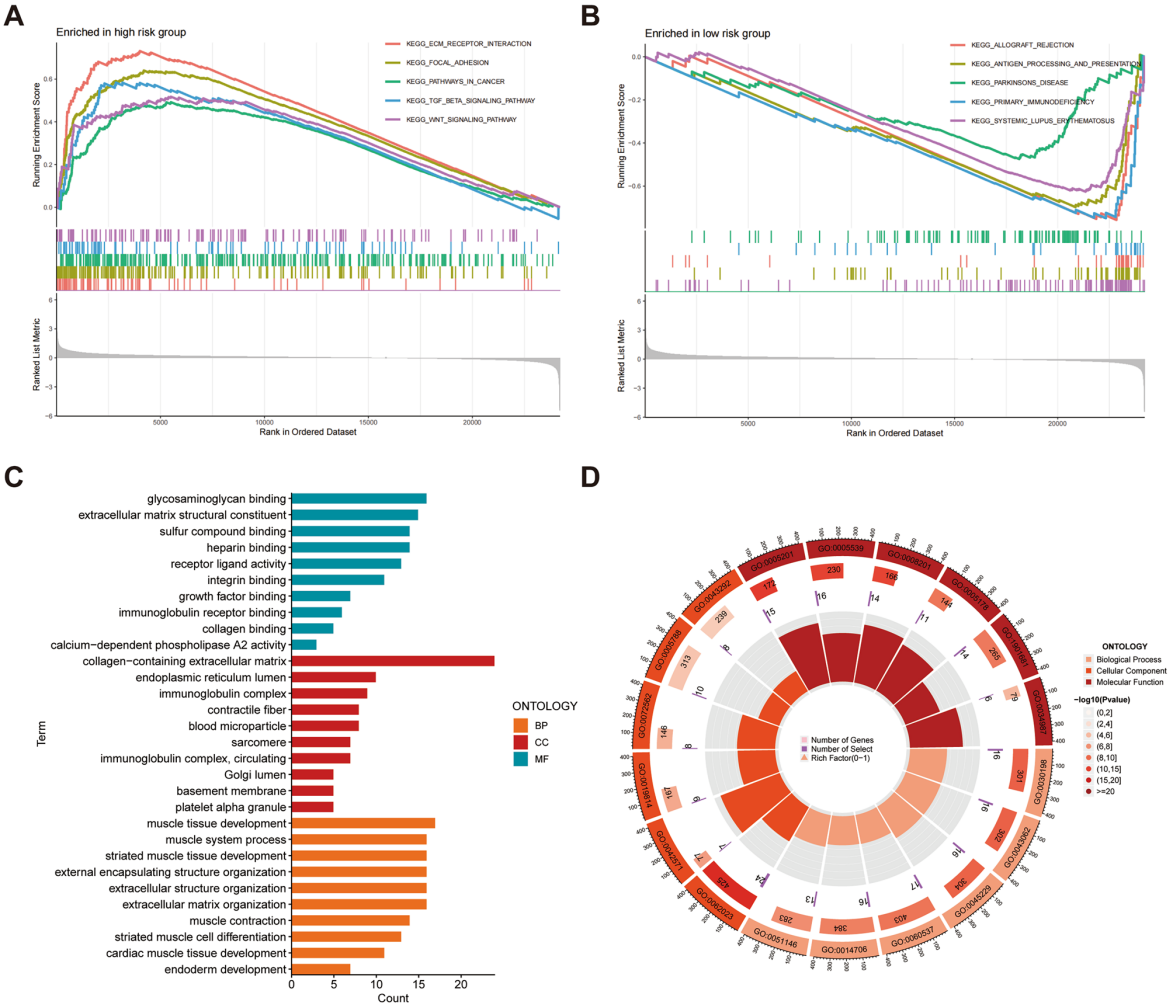
CAFRI-based GSEA and GO analysis. (**A**, **B**) GSEA revealed the five pathways with the highest enrichment in the different risk subgroups. (**C**, **D**) GO analysis to determine the enrichment of DEGs in cellular components, molecular functions and biological processes.

GSVA analysis showed that pathways enriched in high-risk subgroup included signalling pathways such as TGF-β, WNT, mTOR, Notch and MAPK, while pathways such as DNA replication, proteasome, primary immunodeficiency and antigen processing and presentation were enriched in the low-risk subgroup (Fig. 8A). Additionally, heatmaps revealed a broad correlation among the expression of CAFRI-related genes and tumour-related signalling pathways (Fig. 8B), suggesting possible crosstalk of these hub genes in different signalling pathways. In addition, GSEA analysis showed the five pathways with the highest enrichment in the high- and low-risk groups, and the results corroborated with GSVA (Fig. 9A, B).

To explore the molecular characteristics of the differences between different risk subgroups, we performed GO enrichment analysis of DEGs between different risk subgroups. The outcomes showed that DEGs were mainly enriched in molecular functions including glycosaminoglycan binding, extracellular matrix structural components, sulphur compound binding and immunoglobulin receptor binding. In addition, DEGs were also enriched in cellular components such as collagen-containing extracellular matrix, endoplasmic reticulum lumen, immunoglobulin complex and contractile fiber. Regarding biological processes, DEGs were mainly enriched in muscle tissue development, external encapsulating structure organization and extracellular matrix organization (Fig. 9C, D).

### Correlation of the CAFRI and TMB

**Figure 10:**
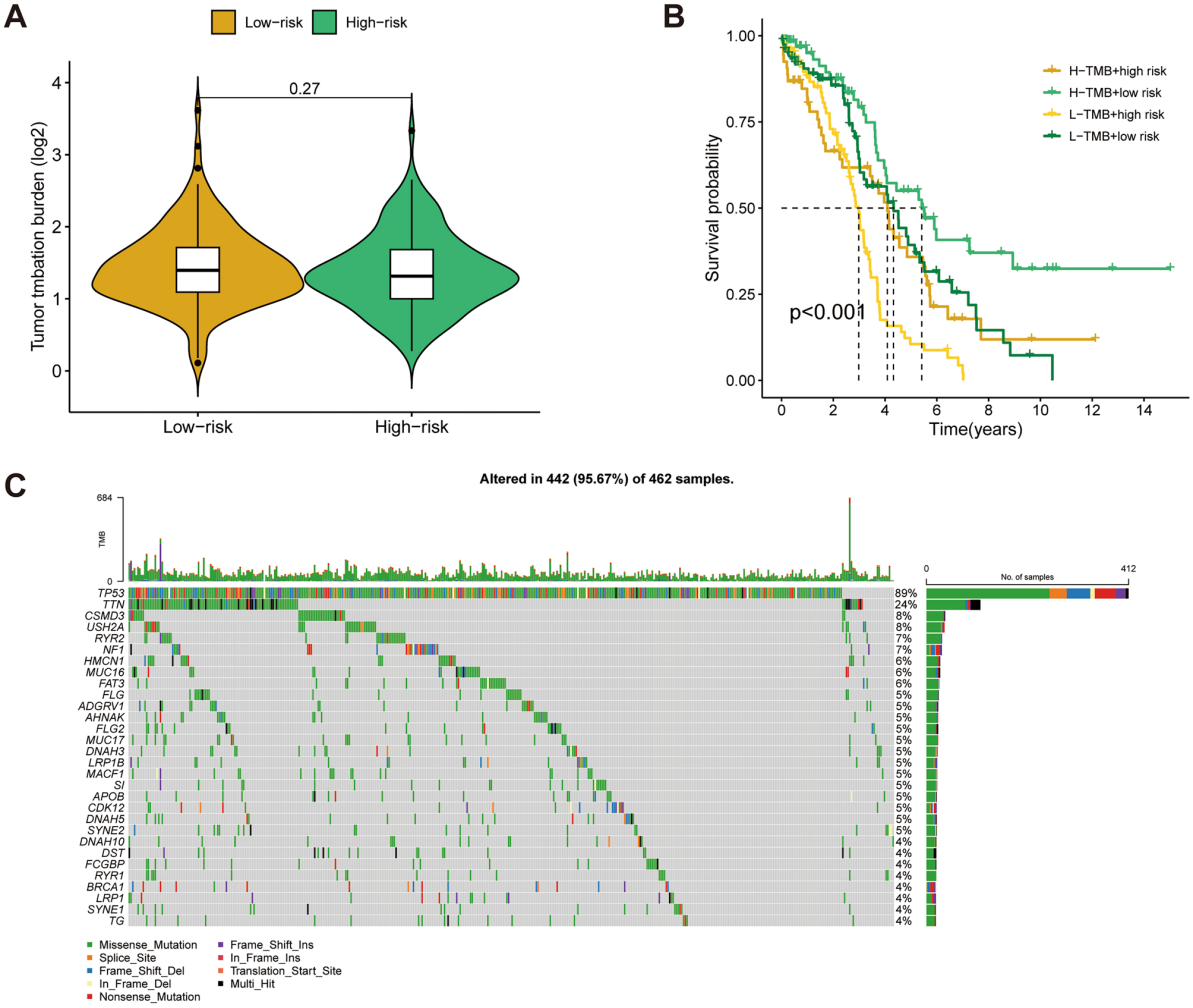
CAFRI-based TMB analysis. (**A**) TMB levels between the high and low risk subgroups. (**B**) Kaplan–Meier curves based on the combination of TMB and CAFRI. (**C**) Mutation waterfall in the TCGA-OV cohort.

Violin plots showed no significant difference in TMB levels between the risk subgroups (Fig. 10A). Nevertheless, K-M analyses showed that the best survival was observed for the combination of high-TMB and low-risk, and the worst survival was observed for the combination of low-TMB and high-risk (*p* < 0.001) (Fig. 10B), indicating that the combination of CAFRI and TMB can be a promising predictor of clinical outcomes in OV. Additionally, waterfall plots showed that *TP53* and *TTN* were genes with high mutation frequencies in OV (Fig. 10C).

### CAFRI predicts the TIME in OV

**Figure 11:**
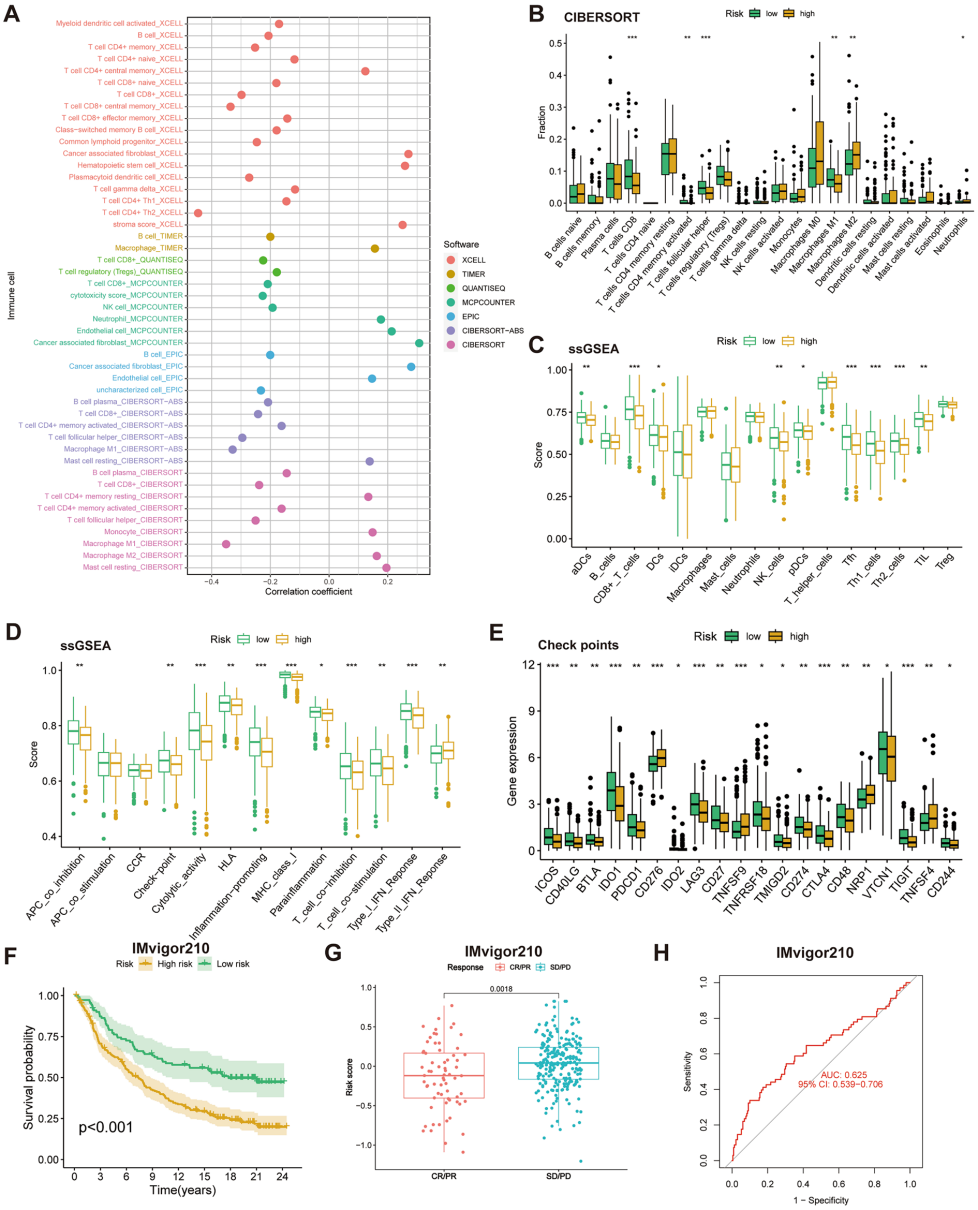
CAFRI-based TIME analysis. (**A**) Six algorithms to determine the relationship between index score and different immune cells infiltration. (**B**) CIBERSORT algorithm identifies differences in the extent of immune cells in different risk subgroups. (**C**, **D**) ssGSEA analysis determines immune cell scores and immune function scores in different risk groups. (**E**) Differences in expression of immune checkpoints. (**F**) Kaplan–Meier curves for CAFRI in the IMvigor210. (**G**) Differences in risk scores between patients with CR/PR and individuals with SD/PD after treatment with ICIs in the IMvigor210. (**H**) ROC curve for CAFRI in the IMvigor210. *P < 0.05, **P < 0.01, and ***P < 0.001.

Considering the regulatory role of CAFs on TIME, we further analysed the correlation between CAFRI and TIME in OV. The EPIC, CIBERSORT, MCPCOUNTER, TIMER, XCELL and QUANTISEQ algorithms of the TIMER 2.0 platform showed that CD8+ /CD4+ T cells and M1 macrophages and other cells were negatively correlated with CAFRI scores, while M2 macrophages were positively correlated with CAFRI scores (Fig. 11A). CIBERSORT results showed that patients in the high-risk subgroup had lower levels of CD8+ T cells, activated CD4+ memory T cells, T follicular helper cells (Tfh) and M1 macrophages. In contrast, M2 macrophages were significantly higher in the high-risk subgroup (Fig. 11B). Further ssGSEA results showed that patients in the low-risk group had significantly higher infiltration of immune cells such as CD8+ T cells, natural killer (NK) cells, Tfh and tumour infiltrating lymphocytes (TIL) (Fig. 11C). Notably, in terms of immune function, immune checkpoints also showed lower levels of expression in the high-risk subgroup (Fig. 11D). Further analyses showed that the majority of immune checkpoints were highly expressed in the low-risk group (Fig. 11E). We further introduced the ICIs treatment cohort IMvigor210 to validate the predictive value of CAFRI for immune efficacy. The K-M curves showed that the IMvigor210 cohort had significantly worse survival in the high-risk group than in the low-risk group (Fig. 11F). In addition, patients in complete response (CR) and partial response (PR) after treatment with ICIs had significantly lower risk scores than those with stable disease (SD) and progressive disease (PD) (Fig. 11G). Furthermore, the ROC curve showed that CAFRI demonstrated high predictive accuracy in the IMvigor210 cohort (AUC = 0.625) (Fig. 11H).

### Application of the CAFRI in clinical treatment

**Figure 12:**
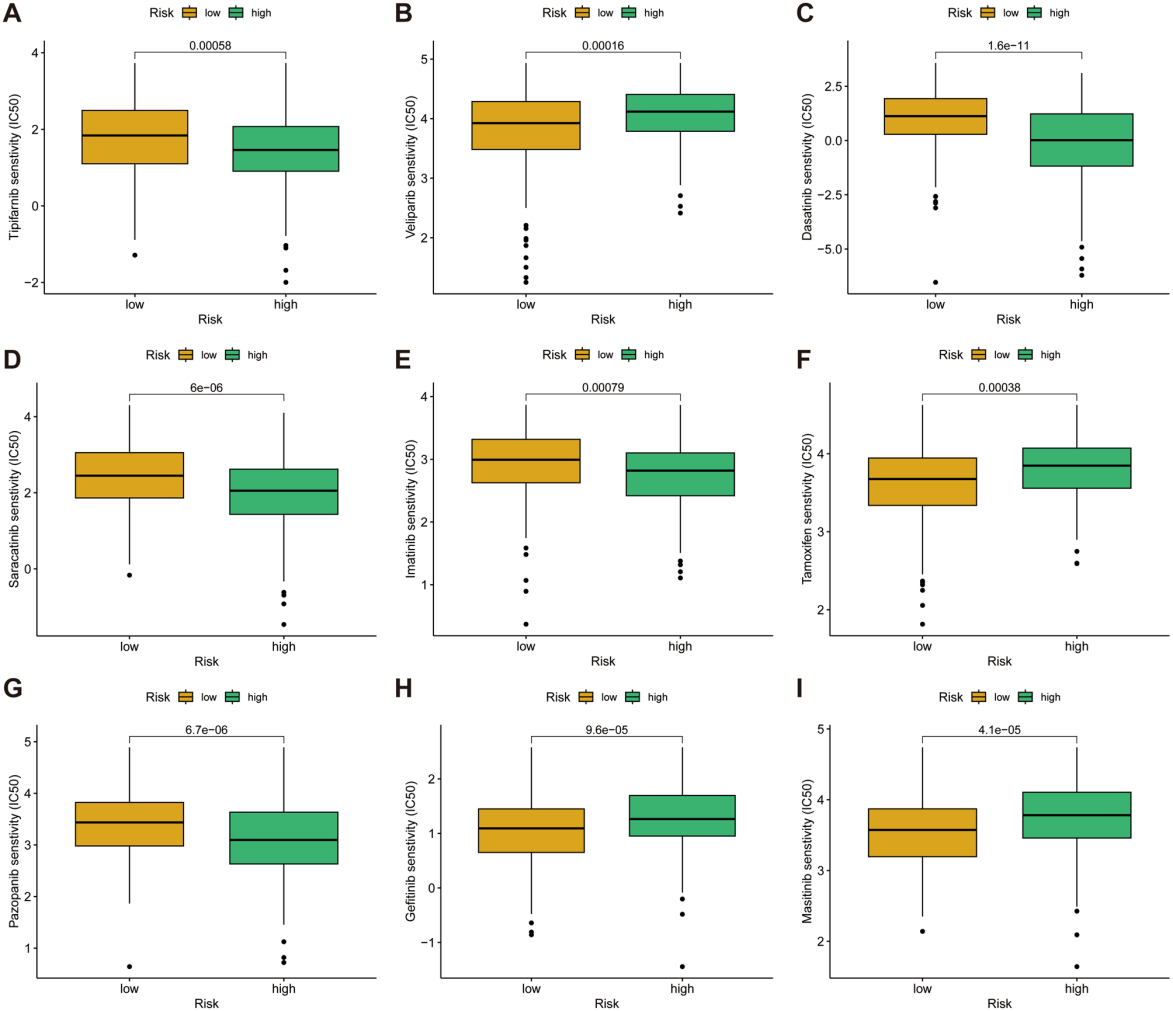
IC50 prediction based on CAFRI. (**A**–**I**) The box plot shows drugs with different IC50 values between the high and low risk groups.

Given the predictive value of CAFRI for prognosis and immunotherapy, we further explored the value of CAFRI in the individualized medication of individuals with OV. Box plots showed that the predicted IC50 values for some clinical treatments differed between the two risk groups (*p* < 0.001) (Fig. 12A–I). Of these, the targeted drugs tipifarnib, dasatinib, saracatinib, imatinib and pazopanib had higher IC50s in the low-risk group. In contrast, veliparib, tamoxifen, gefitinib and masitinib had higher predicted IC50 values in the high-risk population.

## Discussion

Accumulating studies have shown that that CAFs are closely involved in tumour prognosis and the treatment efficacy^38–41^. Targeting CAFs has been shown to restore anti-tumour immunity^42^ and improve the effectiveness of immunotherapy^43^. Previous studies have demonstrated that crosstalk between CAFs and OV affects tumour cell stemness^44^, promotes chemoresistance^45^, and plays a crucial role in the spatial distribution of immune cells in differentiated TME^22^. Furthermore, several studies have confirmed the link between tumour-infiltrating immune cells and clinical outcome in patients with OV^46–48^. Therefore, exploring the potential role of CAFs in evaluating clinical outcome, TIME landscape and effectiveness of immunotherapy in individuals with OV will facilitate the identification of valid biomarkers and therapeutic targets.

Previous studies have shown that the identification of molecular subtypes of OV not only improves our understanding of the molecular basis of OV, but also helps to identify potential therapeutic targets^49^. In this study, we developed a CAFRI containing 13 CAFs-related genes based on an integrated analysis of scRNA-seq and bulk RNA-Seq. Cox regression analysis suggested that the CAFRI-based risk score was an independent prognostic factor for patients with OV. Notably, the stability of the CAFRI was further validated in multiple verification cohorts. As patient age is also an independent prognostic indicator for OV, to better predict the prognosis of patients with OV, we created a nomogram based on the CAFRI that included the patient's risk status and age. The calibration curves showed that the predicted results of the nomogram were in high agreement with the actual results. All results suggest that the CAFRI and nomogram developed in this study are reliable predictive tools for prognosis of OV.

In the last decade, immunotherapy, represented by ICIs, has opened a new epoch of antitumour therapy^50^. However, the low clinical efficiency of immunotherapy is a bottleneck problem in the treatment of ICIs. Studies have confirmed that one of the main reasons for the low response rate of immunotherapy is the lack of infiltration of effector immune cells in the TME, which is referred to as immune ‘cold tumour’^51^, which limits the response of tumours to ICIs. In contrast, immune 'hot tumours' are a category of tumours characterised by immune checkpoint activation and massive infiltration of CD8+ T cells, and are referred to as immunoinflammatory tumours^52,53^. The intrinsic characteristics of TME in ‘hot tumours’ allow for a better response to ICIs^54^. In the present study, CAFRI-based risk stratification showed higher levels of CD8+ T-cell in the low-risk subgroup. Additionally, immune checkpoints, including PD1, PD-L1 and CTLA4, were expressed at high levels in the low-risk subgroup. Together, these outcomes suggest that the low-risk subgroup identified by CAFRI are more likely to be "hot tumours" and may respond better to treatment with ICIs compared to the high-risk population.

Tumour-associated macrophages (TAMs) are an important cell type in TIME and interact closely with CAFs^55,56^. Inflammatory CAFs in TIME play a role in the transition of macrophages to an immune-compromised state by promoting macrophage polarisation from the M1 to the M2 phenotype, which in turn promotes an immunosuppressed state^57^. M1 macrophages, an important subtype of TAM, can kill tumour cells by producing pro-inflammatory factors and reactive oxygen species^58^, whereas M2 macrophages promote tumour progression by suppressing anti-tumour immunity^58,59^. Meanwhile, M2 macrophages have been shown to inhibit the function of ICIs^60^. Infiltration of M2 macrophages often predicts a poor prognosis for OV. Recent studies have shown that reprogramming the TAM phenotype to polarize M2 macrophages into M1 macrophages can reshape the tumour immune microenvironment and has been suggested as a potential strategy to increase the response rate to ICIs^61^. In this study, the low risk population of OV identified by index had higher levels of M1 macrophage infiltration, while M2 macrophages revealed lower levels of infiltration in the low risk population. This result further suggests that the low-risk population may have a higher response rate to treatment with ICIs. To further validate the above findings, we risk-stratified and ICIs efficacy-stratified patients in the immunotherapy cohort IMvigor210. The results were consistent with expectations, with the prognosis of the low-risk subgroup treated with ICIs being significantly better than the high-risk subgroup. Also, risk scores were significantly lower in CR/PR individuals than in SD/PD patients after treatment with ICIs.

The advent of polyADP-ribose polymerase (PARP) inhibitors has revolutionised the treatment paradigm for OV. PARP inhibitors cause single-strand breaks in DNA that accumulate and convert to double-strand breaks^62^. These DNA damages in homologous recombination repair-deficient (HRD) tumours lead to synthetic lethality^62^. Veliparib is an oral PARP inhibitor that has shown efficacy in clinical trials as a single agent and can be used in combination with standard chemotherapy regimens^63,64^. In a phase 3 randomised controlled clinical trial involving previously untreated patients with advanced OV, veliparib concomitantly with chemotherapy and continued as maintenance therapy significantly prolonged progression-free survival (PFS) compared to induction chemotherapy without veliparib maintenance therapy^65^. In the present study, the IC50 values for veliparib were significantly higher in the high-risk subgroup, suggesting CAFRI as a potential complementary tool to predict the benefit of veliparib. Additionally, pazopanib is an oral small molecule tyrosinase inhibitor (TKI) specific for vascular endothelial growth factor receptor (VEGFR) and platelet-derived growth factor receptor (PDGFR). In a phase II clinical trial of recurrent ovarian cancer conducted by Friedlander et al., pazopanib showed good monotherapy activity^66^. The National Comprehensive Cancer Network clinical guidelines currently recommend pazopanib for the treatment of platinum-resistant recurrent OV. In this study, the IC50 values for pazopanib were significantly higher in the low-risk subgroup, indicating that the high-risk subgroup is more likely to be the population to benefit from pazopanib.

Although we have used different cohorts and different algorithms to systematically verify and assess the constructed CAFRI, the study still has certain limitations. Firstly, the present research fails to assess the bias of the dataset in the retrospective analyses. Secondly, the predictive effect of CAFRI on clinical prognosis and drug sensitivity of OV remains to be further validated in a large sample of prospective clinical trials. Furthermore, the key genes in CAFRI are not specifically expressed genes for CAFs, and their regulatory mechanisms for CAFs in the OV tumour microenvironment deserve further exploration.

## Conclusion

The CAFRI developed in this study is a promising predictor of clinical outcomes in patients with OV. In addition, population stratification based on CAFRI can effectively classify individuals with different TIME landscape and assist in determining immune ‘hot tumours’ and ‘cold tumours’. These results provide new insights for the development of biomarkers and the selection of personalised treatment regimens in OV.

### Supplementary Information

Supplementary Figure S1.

## Data Availability

The datasets used and/or analysed during the current study available from the corresponding author on reasonable request.
