# Supplementary material for: Identification of a cancer associated fibroblasts-related index to predict prognosis and immune landscape in ovarian cancer
Source: Sci Rep. 2023 Dec 7;13:21565. doi: 10.1038/s41598-023-48653-w (PMC10700659; doi:10.1038/s41598-023-48653-w)
Supplement: Supplementary file 1 — Supplementary Figure S1. [file 41598_2023_48653_MOESM1_ESM.docx]

Supplementary Material

# 1.Supplementary Figures


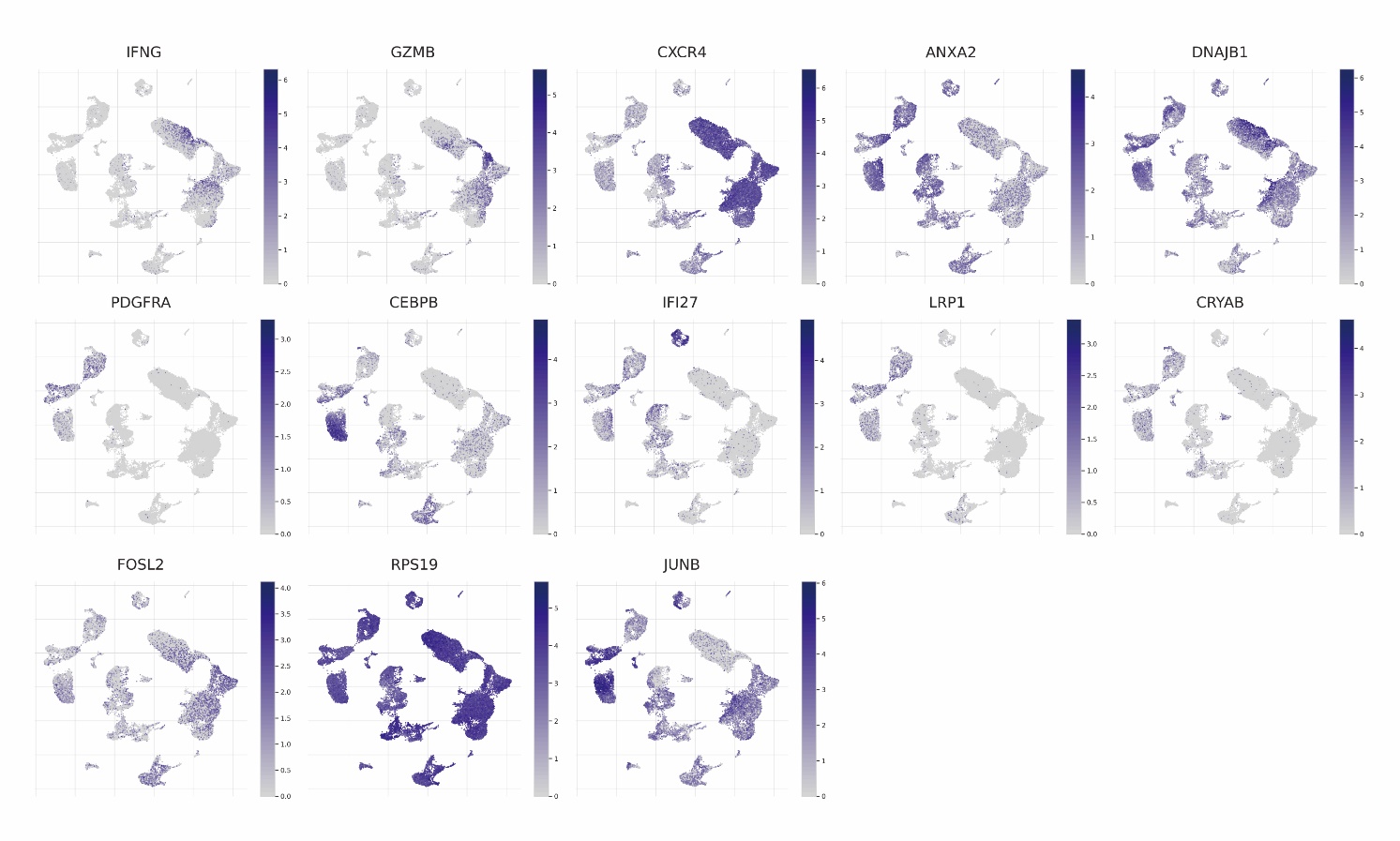


**Figure S1. The expression of the 13 index-related genes in different cells of OV tissue**.
